# Identification of Secondary Metabolites of Interest in *Pleurotus djamor* Using *Agave tequilana* Bagasse

**DOI:** 10.3390/molecules28020557

**Published:** 2023-01-05

**Authors:** Byanka A. Cruz-Moreno, Ana Angélica Feregrino Pérez, Juan Fernando García-Trejo, Sergio Alfonso Pérez-García, Claudia Gutiérrez-Antonio

**Affiliations:** 1 División de Estudios de Posgrado, C.A. Bioingeniería Básica y Aplicada, Facultad de Ingeniería, Universidad Autónoma de Querétaro, C.U. Cerro de las Campanas S/N, Colonia Las Campanas, Santiago de Querétaro 76010, Querétaro, Mexico; 2 Centro de Investigación en Materiales Avanzados S.C. (CIMAV), Unidad Monterrey, Autopista Monterrey-Aeropuerto Km 10, Alianza Norte 202, Apodaca 66628, Nuevo Leon, Mexico

**Keywords:** agro-industrial residues, agave bagasse, mushrooms, bioactive compounds, metabolites, antioxidant capacity

## Abstract

Agro-industrial residues represent more than 60% of organic wastes worldwide, which could be used to generate other by-products or to be incorporated into other production chains. For example, bagasse is a waste from the tequila industry in Mexico that could be implemented for mushroom cultivation. Additionally, the substrate influences the growth, development, and production of secondary metabolites of fungi. This work presents a comparative experiment that studies the metabolite production in *Pleurotus djamor* mushrooms on agave bagasse and barley straw (traditional substrate). The biological efficiency (BE), yield, phenolics and flavonoids, antioxidant capacity, tannins, and the identification of low molecular weight metabolites were evaluated. Five treatments were proposed according to the following mixtures of agave bagasse: barley straw: T1 (1:0), T2 (3:1), T3 (1:1), T4 (1:3), and T5 (0:1). T2 had the highest yield (13.39 ± 3.23%), BE (56.7 ± 13.71%), and flavonoids (44.25 mg rutin equivalent (RE)/g); T3 obtained the highest phenol content (230.27 mg GAE/g); and T1 the highest tannins content (0.23 mg (+) catechin equivalent (CE)/g). Finally, T1 and T5 are the ones that present the greatest number of primary metabolites, including hydroxycitric acid, 2-deoxy-D-galactose, D-mannose, paromomycin, palmitic acid, pyrrole, mannitol, and DL arabinose, while in T2, T3, and T4 only two chemical compounds were found present (palmitic acid and pyrrole in T2, silicic acid and pyrrole in T3 and 2-deoxy-D-galactose and quinoline in T4). The cultivation substrate influences the concentration of bioactive molecules in the fruiting bodies of *P. djamor*. Additionally, *P. djamor’s* degradation of agave bagasse residue generates a potential application for agro-industrial residue management at a low cost.

## 1. Introduction

*Pleurotus djamour* is a pantropical fungus that belongs to the Agaricales group with a high medicinal and commercial value and protein content. This mushroom has a short growth period compared to other edible species. It can be cultivated easily and cheaply through a process known as solid fermentation [1]. These edible mushrooms have a high gastronomic value and are considered a functional food because of their high nutritional and medicinal properties [2,3,4,5]. Different investigations have shown their high content of proteins, carbohydrates, minerals (calcium, copper, phosphorus, magnesium, potassium, and zinc), vitamins (riboflavin (B2), niacin (B3), provitamin (D), and dietary fibers (D-glucans, chitin and pectic substances); and low-fat content [6]. Therefore, they are a high-potential product whose international market is estimated at USD 34 billion [7]. The culture substrate has a great influence on mycelial growth and the formation of basidiomas (fruiting bodies) since it provides the nutrients so that the fungus can complete its life cycle. Therefore, the quality of the fruiting bodies, both physical and nutritional, is directly related to the quality of the substrate on which it develops [8].

Lignocellulosic fungi, such as those belonging to the Pleurotus genus, grow favorably in agricultural and forestry residues containing a high percentage of lignin *P. djamor* has been cultivated on different substrates, mostly on agro-industrial residues, with acceptable biological efficiency (BE) results [8,9,10,11,12]. 

*P. djamor* culture on barley straw is common, with known BE values. However, studies of the cultivation of this fungus on *Agave tequilana* bagasse have not yet been reported. Agave bagasse (AB) is an agricultural residue available throughout the year due to the importance and constant growth of the Mexican tequila industry. Every year, an average of 600 tons of this waste is produced. Usually, this waste is composted, but the reintegration time is long (8–20 months) compared to how quickly it is generated (Tequila Regulatory Council 2021); therefore, a revaluation or adequate final disposal is urgent. On the other hand, barley straw (BS) is continuously produced, accessible, and cheap. The use of AB for mushroom production would provide a low environmental impact solution for its treatment, thereby avoiding the pollution problem associated with its slow degradation and inadequate disposal. This work aimed to study the yield, chemical composition, as well as the nutritional and nutraceutical value of *P. djamor* grown on a mixture of *Agave tequilana* bagasse and barley straw at different proportions.

## 2. Results and Discussion

### 2.1. Performance and Biological Efficiency

The days the mycelium takes to colonize the substrate is an important parameter in mushroom production. A higher proportion of agave bagasse reduced the days for the mycelium to colonize the substrate (8 days). There was a statistical difference between the highest and lowest percentages of agave bagasse with the control. Optimal mycelial colonization is necessary to obtain the maximum yield (r) of fruiting bodies. As observed in the results, the highest yield percentage coincides with fewer days to get colonization by the mycelium (Table 1). T2 presented the highest yield and T5 the lowest.

The biological efficiency (BE) and the primordial formation of the substrates are shown in Table 2. *P. djamor* reached its highest BE in T2, which is a mixture of agave bagasse substrate with barley straw (3:1) and coincides with what has been pointed out by some authors: yield and BE increase when using mixtures of substrates in comparison with pure substrates [8,10,13,14]. On the other hand, the lowest biological efficiency is observed in T1, which is a pure substrate of Agave bagasse; in addition, there is a statistically significant difference between treatments as shown in Table 2. These results agree with those reported by [8,15,16], with BE values from 24% to 123.29%. Results presented with the mean of each triplicate per experiment with SE. Means with different letters in the same column are statistically different (T-Student α ≤ 0.05).

**Figure 1:**
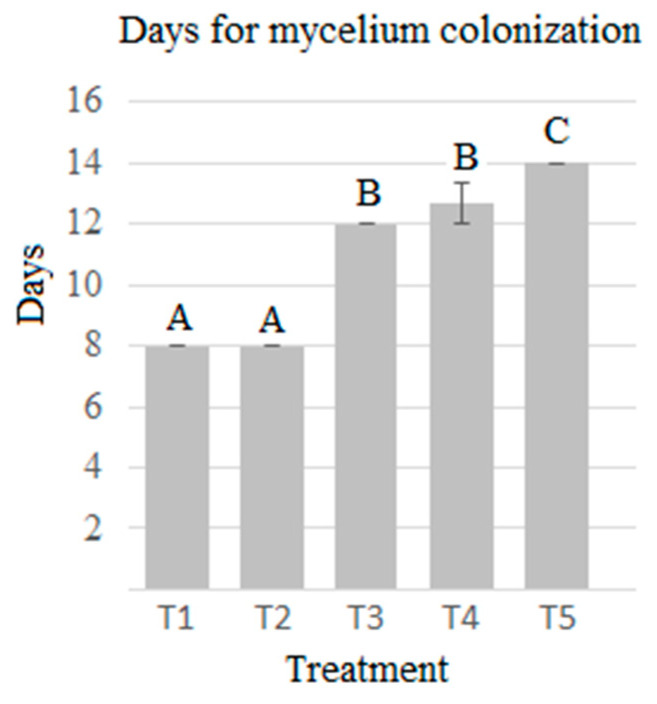
Colonization in *P. djamor* over the five different treatments. The bars indicate the mean of each experiment in triplicate with SE Different letters indicate significant difference (T-Student *p* ≤ 0.05).

Mycelial growth and primordia formation were faster in T1 and T2, while the slowest development occurred in T5 (Figure 1). There was a statistically significant difference in at least one of the treatments for mycelial growth (ANOVA, *p* < 0.05), but no statistically significant differences were found in the primordia formation. These results were superior to those reported by [17,18,19,20] whose mycelia completely covered the substrate in an average of 18 days.

### 2.2. Bromatological Analysis of Fruiting Bodies (Basidiomycetes)

Species of the genus Pleurotus use lignin, hemicellulose, and cellulose as food sources, secreting exoenzymes to degrade the substrate into simpler molecules to obtain the necessary nutrients for their development. Changes in the fungus nutrition will be observed depending on the substrate content. Table 2 shows the results of the bromatological analysis performed on the fruiting bodies of *P. djamor*. T5 obtained the highest humidity content, attributable to the high water retention capacity of the substrate [21]. T1 presented the lowest moisture content, attributable to the poor water retention capacity of the substrate, compared to the others. These values were similar to [22], who reported 89.71–91.56% for various agro-industrial wastes. Similarly, refs. [6,23,24,25,26] reported values from 83.3 to 85% for various Pleurotus species cultivated in different agro-industrial residues. Results presented with the mean of each triplicate per experiment with SE. Means with different letters per column are statistically different (T-Student α ≤ 0.05).

For all treatments, the lipid content does not exceed 1%. These values are below the ranges reported for Pleurotus, from 0.20 to 3.10 [2,27,28]. T1 and T5 presented the highest lipid content, while T4 had the lowest. Fatty acids and triterpenoids are present in this mushroom. The presence of triterpenoids makes this fungus a nutraceutical food [28]. The ash content represents the composition of mineral elements. The results are within the range reported by different authors (1–7.9%) [27,29,30], increasing from T2 to T5, attributable to the substrate composition. In fungi, carbohydrates are mainly presented as sugars that serve as a primary energy source and for chitin and hemicellulose generation [31]. In this experiment, carbohydrates were the most abundant macronutrients, with values ranging from 91.67% (T5) to 95.88% (T1), above the reported by [2,27,32] (44.0–81.8%). The protein content was 2.69–6.50%, within the range reported by different authors (4–11%) [30,33,34,35,36] and below the value reported by [37] of 17.92%. In this context, Jaworska et al. [27], reported that the substrate’s quantity and type of proteins influence the protein content of the fruiting bodies.

It is known that the content of proteins, carbohydrates, lipids, and ashes as well as other nutrients found in the fruiting bodies of mushrooms is influenced by the substrate on which it develops, for which it is important to know the content of these nutrients present in the substrate used [38].

### 2.3. Quantification of Phenols

**Figure 2:**
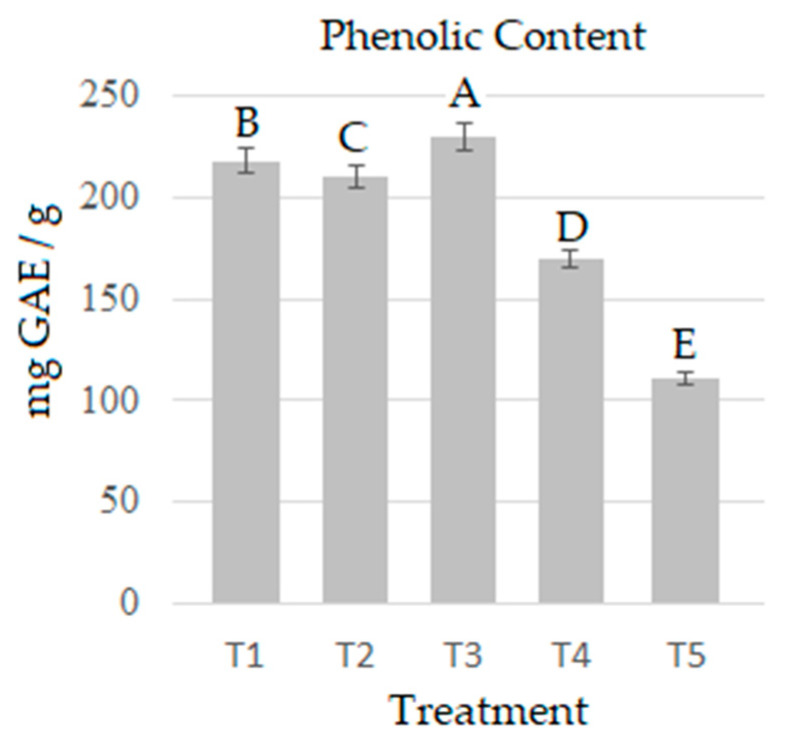
Phenolic content in *P. djamor* over the five treatments. The bars indicate the mean of each treatment in triplicate with SE. Different letters indicate significant difference (T-Student *p* ≤ 0.05).

Figure 2 presents the phenolic content (mg GAE/g) of *P. djamor* per treatment. The highest content was identified in T3 (230.27 mg GAE/g), followed by T1 (218.37 mg GAE/g). Studies have found that the nature and chemical composition of the substrate where mushrooms grow affects their polyphenolic and antioxidant profile [33]. Phenolic contents in this study were superior to those reported by [5,39,40,41,42,43] with values of 0.5–1.768 mg GAE/g that include different species of mushrooms (*P. ostreatus*, *P. citrinopileatus*, *Boletus edulis*, *Cantharellus cibarius*, *Craterellus cornucopioides*, *Calocybe gambosa*, *Hygrosphorus marzuolus*, and *Lactarius deliciosus*) growing on different substrates except Agave tequilana bagasse. The substrate influenced the production of total phenols, and this particular species showed a high content in all treatments. Phenols and flavonoids are secondary metabolites of greater pharmacological relevance present in the fruiting bodies of Pleurotus sp [44]. There is a positive correlation between the antioxidant activity and phenolic content of extracts of edible medicinal mushrooms [45]. Fungal extracts of Pleurotus are potential candidates for obtaining compounds of interest for human health and for developing valuable products in order to treat diseases related to oxidative stress.

### 2.4. Condensed Tannins

**Figure 3:**
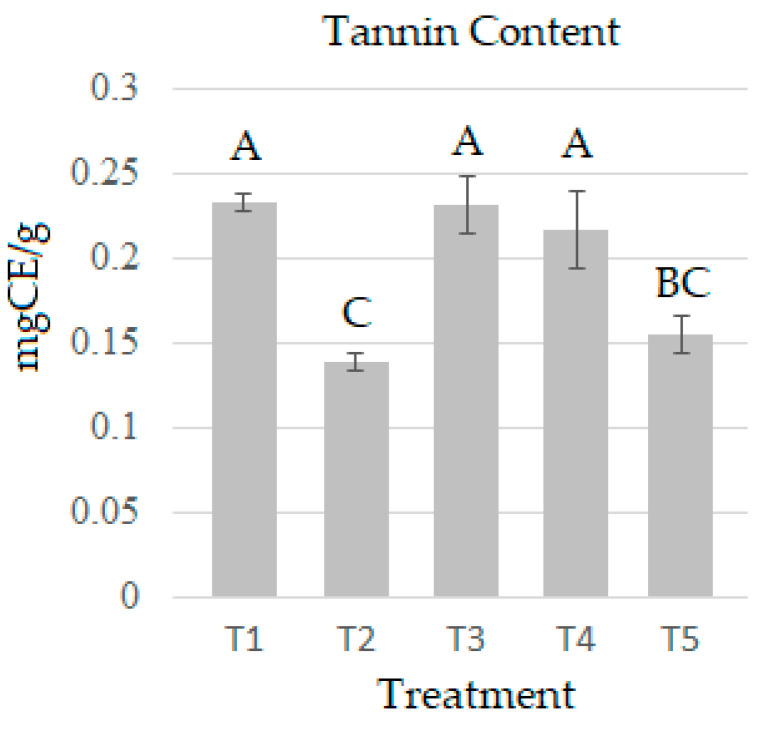
Tannin content in *P. djamor* over the five treatments. The bars indicate the mean of each experiment in triplicate with SE. Different letters indicate significant difference (T-Student *p* ≤ 0.05).

Tannins are antinutritional components present in edible mushrooms. T1 presented the highest value (0.2328 mg CE/g), and T2 had the lowest (0.1386 mg CE/g), with a statistical difference between all treatments (Figure 3). These levels are more than 90 times below the safe limit the World Health Organization prescribes. The results of this study are lower than those reported by [43] (3.691 ± 0.011 CE mg/g) for *P. ostreatus* grown on sawdust and those reported by [46] for Ganoderma (2.29 ± 0.24 mg/g) but above the values reported by [47] for *P. ostreatus* and *Agaricus bisporus* with values of 0.0077–0.0155 mg/g. The results of condensed tannins of this experiment are the first reported for *P. djamor* cultivated on barley straw and Agave bagasse.

### 2.5. Quantification of Flavonoids

**Figure 4:**
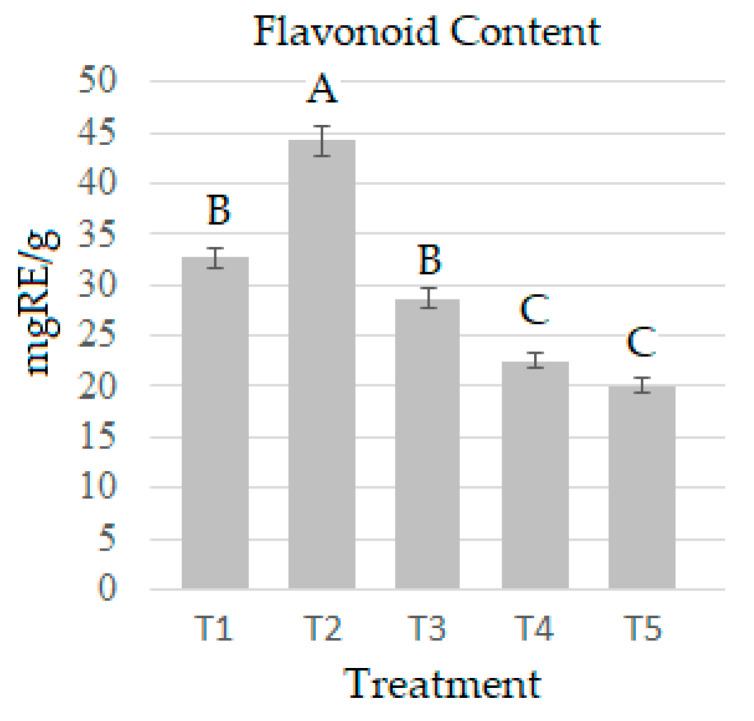
Flavonoid content in *P. djamor* over the five treatments. The bars indicate the mean of each experiment in triplicate with SE. Different letters indicate significant difference (T-Student *p* ≤ 0.05).

The concentration of flavonoids varies according to the treatment and does not correlate with the phenolic concentration. T2 (44.252 mg RE/g) and T1 (28.668 mg RE/g) had the highest amount of total flavonoids (Figure 4), while T4 and T5 had the least content. The flavonoid content recorded for other Pleurotus varieties ranges between 0.130 and 0.307 mg QE/g in *P. ostreatus* [39,41,46], and 0.17 ± 0.02 mgQE/g for *P. florida* [48]. Additionally, these values were obtained by taking Quercetin as a reference standard instead of Rutin (both compounds are used as a reference in the quantification of total flavonoids). The results of this variable presented in this study are the first for *P. djamor* cultivated in barley straw and agave bagasse. Besides the substrate, other environmental factors may also influence the production of different classes of bioactive compounds [5].

### 2.6. Determination of the Antioxidant Capacity by the DPPH Method

**Figure 5:**
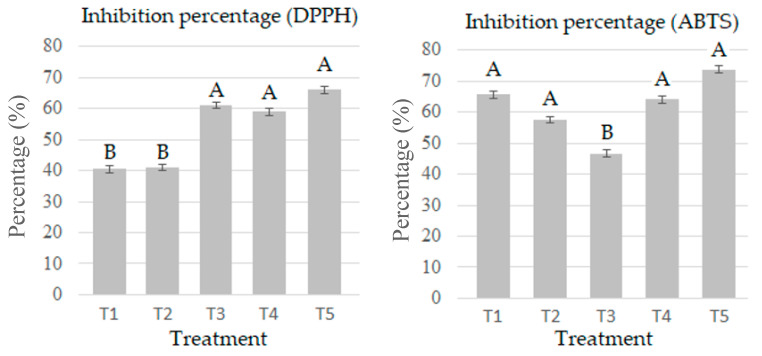
Inhibition percentage by DPPH and ABTS in *P. djamor* in the five treatments. The bars indicate the mean of each experiment in triplicate with SE. Different letters indicate significant difference (T-Student *p* ≤ 0.05).

Mushrooms are a rich dietary source of antioxidants. The consumption of food with a high content of antioxidants is associated with reductions in oxidative stress-related diseases and disorders [48] (Wong et al., 2013). Figure 5 shows the percentage of DPPH inhibition of the fruiting bodies of *P. djamor* grown on the different substrates. Statistical differences were observed between the treatments with the highest percentage of Agave bagasse and those with the lowest percentage.

### 2.7. Determination of the Antioxidant Capacity by the ABTS+ Method

Figure 5 shows the antioxidant capacity values by the ABTS method and the percentage of ABTS inhibition of the fruiting bodies of *P. djamor* grown on the different substrates. The ability to scavenge the ABTS+ cationic radical ranged from 166.8604 to 221.0797 μM Trolox/g. Other studies showed lower antioxidant capacity, as in [41], with values of 27.17 μM Trolox/g. The results of this variable may be due to the harvest stage, climate, soil, and different chemical composition [39]. T1 and T5 presented the highest percentage values of inhibition by ABTS; hence, the mushrooms grown in these substrates have the potential to be used as natural antioxidant sources.

### 2.8. Gas-Chromatography-Mass Spectrometry (GC-MS) Analysis

Multiple investigations have been performed on the different chemical compounds found in edible mushrooms due to their nutraceutical properties (e.g., antioxidant, anticholesterolemic, and antitumor capacity [2,3,4,5,6]. This work identified nine compounds (in addition to the metabolites mentioned above) (Table 3). Pyrrole (1, 2, a) pyrazine 1, 4, dione, hexahydro 3-(2-methylpropyl), 2-deoxy-D-galactose, and palmitic acid were the main compounds found in the analyzed fungal samples. Some compounds were found in more than one treatment, such as palmitic acid and 2-deoxy-D-galactose in T1, T2, and T5. Others only appeared in one treatment, such as hydroxycitric acid and paromomycin in T1, silicic acid in T3, quinoline in T4, and mannitol in T5. These results differ from those reported by [37,49], which include: 4-hydroxybenzoic acid, 5-feruloylquinic acid, p-hydroxybenzoic acid, hydroxyphenolic acid, protocatechuic acid, chlorogenic acid, and myricetin that were not detected in this work. However, our results show that the strain contains compounds with therapeutic applications, such as the commonly used antibiotics paromomycin and quinoline, the anticancer metabolite pyrrole, and the tumor growth inhibitor 2-deoxy-D-galactose. Pyrrole [1,2-a]pyrazine-1,4-dione, hexahydro-3-(2-methylpropyl)- or cyclo(-Leu-Pro) is a compound with anticancer characteristics since it induces apoptosis in cell lines of cancer (A549 and HeLa) without affecting normal cells [50]. The presence of unsaturated fatty acids (i.e., oleic acid, palmitic acid, and hydroxycitric acid) constitutes a favorable nutraceutical characteristic since they protect against cardiovascular diseases and arteriosclerosis caused by cholesterol [51]. In particular, hydroxycitric acid is known for decreasing the synthesis of fatty acids and lipogenesis that induces weight loss [52]. This compound is traditionally extracted from the *Garcinia cambogia* plant [53], and this study is the first record in *P. djamor*.

Finally, all the mentioned results show that the substrate on which *P. djamor* mushrooms are grown influences their nutritional and nutraceutical value, as shown by [14,38,54]. Nevertheless, this study does not show the nutritional or antioxidant content of the substrates used, the results found in the 5 treatments are different, as acknowledged by the mentioned authors.

## 3. Materials and Methods

### 3.1. Location and Cultivation System

The experiment was performed at the Universidad Autonoma de Querétaro, Facultad de Ingenieria, Campus Amazcala, which is located in the municipality of El Marques, Queretaro, México (lat. 20°73′ N, long. 100°26′ W, 1920 m.a.s.l.). Agave tequilana bagasse was obtained from a tequila production plant in Guanajuato state and was exposed to direct sunlight for five days. Barley straw was acquired from Forrajes El Crucero company and was ground in a mill, reaching 5–10 cm pieces. Five substrate mixtures (treatments) were prepared according to Table 1. Polypropylene bags of 50 × 70 cm were filled with 500 g of dried mixtures and placed in a 70 L container with water for hydration for 24 h. Subsequently, they were sterilized for two hours at 100 °C and allowed to cool at room temperature. The substrate was inoculated under aseptic conditions, cleaning the surfaces with 10% chlorine solution and alcohol burners. Fifty grams of inoculum (wheat seed with fungus mycelium) and 5 g of calcium carbonate were added to each bag with manual distribution. Three replicates were elaborated for each treatment and strain. The inoculated bags were incubated at room temperature of 25 ± 5 °C and a relative humidity range of 50–70% in a dark room until the mycelium completely covered the bags. Each bag represented an experimental unit. Once the mycelium completely covered the substrate, they were moved to a growing zone, consisting of a dark room with a relative humidity of around 70%. After primordia formation, several cuts of approximately 2 cm were made on the bag surface to allow fruiting development.

### 3.2. Sampling and Analysis Methods

Bag total colonization and primordia formation was recorded per day. Each value represents the mean of each triplicate experiment. The fruiting bodies of the first three shoots were harvested and weighed when the edge of the cap mushroom was fully extended. Yield (r) (Equation (1)) and biological efficiency (BE) (Equation (2)) were determined in the three harvests.

**r = (mushroom fresh weight/wet substrate weight) × 100** (1)

**BE = (mushroom fresh weight/Dry substrate weight) × 100** (2)

The experiment was established according to a completely random design. Data were subjected to an analysis of variance using GraphPad Prism 7 software.

### 3.3. Bromatological Test of Fruiting Bodies

The following tests were performed in triplicate for the sample analysis: gravimetric method to determine ashes percentage, moisture percentage, and total dietary fiber, microwave for total fats, digestion, and spectrophotometry to determine total protein percentage, isoperibolic calorimeter to determine calories, and spectrophotometry to quantify total carbohydrates.

### 3.4. Methanolic Extraction for Polyphenolic Compounds

Methanolic extractions were performed as described by [55], where 200 mg of sample was placed, and 10 mL of pure methanol (99.98% HPLC grade) was added to each sample. They were kept free of light and shaken for 24 h. Centrifuging (Thermo Scientific, Waltham, MA, USA) at 5000× *g* rpm for 10 min at 4 °C, the pellet formed at the bottom was eliminated, leaving the supernatant.

### 3.5. Quantification of Total Phenols

Total phenolic content was determined using the Folin–Ciocalteu method [56] with modifications. To 40 μL of extract (supernatant) were added 460 μL of distilled water, 250 μL of Folin–Ciocalteu reagent, and 1250 μL of 20% sodium carbonate solution. After 2 h in the dark samples were read at 750 nm in a UV/V is spectrophotometer (Genesys 10S UV-Vis, Thermo Fisher Scientific, Waltham, MA, USA). The quantification was performed by interpolating the results into a gallic acid standard curve (0 to 20 mg). Each extract was analyzed in triplicate, and the results were expressed as mg gallic acid equivalent (GAE) per gram of sample (mg GAE/g).

### 3.6. Condensed Tannins

The quantification of condensed tannins was performed per triplicate following the procedure described by [57] using a 96-well microplate. Fifty microliters of the methanolic extract was placed in a microplate with 200 µL of the 1:1 (*v*/*v*) solution of HCl 8%: vanillin 1%. For the blank, 50 µL of the sample was placed, and 200 µL of 4% HCl was added. Condensed tannins were quantified in a MULTISKAN ASCENT plate reader with 492 nm filters. The results were expressed as mg (+) catechin equivalent per gram of sample (mgCE/g).

### 3.7. Total Flavonoids

Total flavonoid content was determined using the method described by [58], which consists of mixing 50 µL of the sample with 180 µL of methanol and 20 µL of 1% 2-aminoethyl diphenyl borate solution in a 96-well microplate. Absorbance was recorded using the MULTISKAN ASCENT plate reader with 404 nm filters. The results were expressed as mg rutin equivalent (RE) per gram of sample (mgRE/g). Each extract was analyzed per triplicate.

### 3.8. Antioxidant Activity

#### 3.8.1. DPPH

Antioxidant activity was determined using the DPPH free radical scavenging assay (DPPH), following the method of [59] modified by [60] for microplate use. The DDPH (diphenyl-picryl-hydrazyl) reagent was prepared in a 25 mL flask, adjusted with methanol, and added to the supernatant resulting from the extraction. Absorbance was measured in a spectrophotometer (Thermo, MULTISKAN ASCENT) at 532 nm every 10 min for 90 min. The plate was covered and kept in the dark at room temperature between readings. A standard curve with Trolox at 0.05–0.8 mM concentrations was used. The antiradical activity (ARA) was calculated by the percentage of DPPH decolorization using Equation (3), where S_abs_ is the absorbance of the sample at 532 nm, and C_abs_ is the absorbance of the control (absence of antioxidant). The Trolox equivalent antioxidant capacity (TEAC) was calculated using Equation (4), where S_abs_ is the absorbance of the sample at 532 nm, m is the slope of the calibration curve, and S_con_ is the concentration of each sample.

**ARA = (1 − S_abs_/C_abs_) × 100** (3)

**TEAC = (S_abs_/m × S_con_)** (4)

#### 3.8.2. ABTS+

The antioxidant activity of the fungal extracts was determined with the method developed by [61], modified for microplate using 2,2′-azinobis (3-ethyl-benzothiazolin,6-sulfonic acid). Potassium persulfate was added to samples at a concentration of 140 mM and protected from light at room temperature for 12 h. The absorbance was measured with an ELISA reader at a wavelength of 734 nm, from 0.7 to 1. Additionally, 19.20 mg of reagent was added to 5 mL of distilled water, and 88 µL of sodium persulfate was added to form the ABTS radical. The samples were prepared in a 96-well microplate with the extract and ABTS+. Methanol and ABTS+ were used as control and a mixture of the sample with methanol as blank. Absorbance was measured at 734 nm in an ELISA reader for 0–90 min. A standard curve using Trolox concentrations from 50 to 800 µM was used. Results are expressed as µM Trolox/g. The ABTS antioxidant capacity was calculated using Equation (5).

**Inhibition % = ((Initial_abs_ − final_abs_)/(Initial_abs)_)) × 100** (5)

#### 3.8.3. Gas-Chromatography-Mass Spectrometry (GC-MS) Analysis

Each sample was prepared in methanol HPLC (1 mg/mL). Methanol was evaporated with nitrogen gas, and 50 μL of derivatizing agent, BSTFA (N,O-bis[trimethylsilyl]trifluoroacetamide, CAS 2561-30-2, Merck KGaA, Darmstadt, Germany) + 1% TMCS (trimethylchlorosilane) (CAS 75-77-4, Wacker Chemie AG, Burghausen, Germany ) was added. The solution was stirred for 2 min at room temperature, and 1 μL of the sample was injected in triplicate in an Agilent gas chromatograph (GC) series 7890A coupled to a single quadrupole mass spectrometer (MS) detector (model 5975C, Agilent Technologies, Waldbronn, Germany) equipped with an electron impact (EI) ionization source. The cattier gas (helium) flow rate was maintained at 1 mL min-1. The injector temperature was set at 250 °C in splitless mode. An HP-5MS capillary column (30 m length, 250 μm of inner diameter, and 0.25 μm of film thickness) was used. The initial oven temperature was 100 °C for 1 min, rising to 220 °C at 6 °C/min, and maintained for 1.23 min. Then, the temperature was increased to 290 °C at 10 °C/min, and finally raised to 310 °C at 40 °C/min, and maintained for 7.5 min. El energy was set at 70 eV, and the mass range was set at *m*/*z* 50 to 700. GC-MS control and data processing were performed with the Chem-Station (Agilent Technologies, Waldbronn, Germany) software version C.01.10.

## 4. Conclusions

The use of agave bagasse as a substrate to grow mushrooms has an influence on the bioactive compounds (e.g., tannins, flavonoids, and phenolic compounds) present in the fruiting bodies. This fungus has the potential to be used as a natural source for the development of medicines and nutraceuticals. Additionally, the tequila bagasse substrate is a substrate that provides the necessary nutrients for better yield, quality, and synthesis of compounds of interest in *P. djamor*, compared to the traditional substrate. The high percentage of waste generated by the tequila industry is an area of opportunity to obtain bioactive compounds and by-products. The final disposal of *Agave tequilana* bagasse is complicated; thus, a revaluation is an option to reduce its environmental impact and benefit the local economy.

## Figures and Tables

**Table 1:** Proportions of agave bagasse: barley straw of the substrates used for the cultivation, yield (r) %, and biological efficiency (BE) %.

Treatment	Agave Bagasse: Barley Straw Proportions	r (%)	BE (%)	Colonization (Day)	Primordial Formation (Day)
T1	1:0	10.867 ± 0.395 ^B^	36.60 ± 1.33 ^B^	8 ± 0 ^C^	14 ± 0 ^B^
T2	3:1	13.39 ± 0.323 ^A^	56.7 ± 1.371 ^A^	8 ± 0 ^C^	15.67 ± 2.88 ^A^
T3	1:1	10.629 ± 0.04 ^B^	51.40 ± 0.28 ^C^	12 ± 0 ^B^	16 ± 0 ^A^
T4	1:3	7.64 ± 0.21 ^C^	40.40 ± 1.11 ^D^	12.67 ± 1.15 ^B^	16 ± 0 ^A^
T5	0:1	7.25 ± 0.197 ^D^	43.73 ± 1.19 ^D^	14 ± 0 ^A^	17.33 ± 2.31 ^A^

The values indicate the mean of each experiment in triplicate with SE. Different letters indicate significant difference (T-Student *p* ≤ 0.05).

**Table 2:** Bromatological analysis of the fruiting bodies of *P. djamor* in 5 different treatments.

	Humidity	Lipids	Total Protein	Ash	Carbohydrates
T1	84.83 ± 0.13 ^C^	0.28 ± 0.01 ^A^	6.50 ± 0.13 ^A^	1.55 ± 0.06 ^A^	91.67 ± 0.2 ^C^
T2	87.13 ± 0.52 ^B^	0.27 ± 0.02 ^A^	5.88 ± 0.12 ^B^	1.58 ± 0.08 ^A^	92.27 ± 0.55 ^C^
T3	88.04 ± 0.05 ^A^	0.17 ± 0.02 ^B^	4.09 ± 0.18 ^C^	1.28 ± 0.04 ^B^	94.46 ± 0.23 ^B^
T4	87.91 ± 0.57 ^B^	0.14 ± 0.02 ^C^	3.67 ± 0.14 ^D^	1.07 ± 0.04 ^D^	94.91 ± 0.7 ^AB^
T5	89.4 4± 0.13 ^A^	0.28 ± 0.01 ^A^	2.69 ± 0.42 ^E^	1.1 5± 0.03 ^C^	95.88 ± 0.3 ^A^

Results presented with the mean of each triplicate per experiment with SE. Means with different letters per column are statistically different (T-Student *p* ≤ 0.05).

**Table 3:** Chemical compounds identified in *P. djamor*.

Treatment	Chemical Compound	Use	CAS	Retention Time (min)	Molecular Weight (g/mol)
T1	Hydroxycitric acid	Limits the transformation of carbohydrates into fats by inhibiting the enzyme ATP-citratolyase.	3530-14-1	3.82	208.123
	Palmitic acid	Saturated fatty acid, common in beauty products.	57-10-3	6.2	256.4
	Paromomycin	Common oligosaccharide in the use of parasitic infections (antibiotic)	1263-89-4	7.807	615.629
	2-deoxy-D-galactose	Tumor growth inhibitor.	1949-89-9	15.5	164.068
	Pyrrole (1, 2, a) pyrazine 1, 4, dione, hexahydro 3- (2-methylpropyl)	Anticancer metabolite.	5654-86-4	17.3	210.273
	D-mannose	Useful sugar in urinary infections.	3458-28-4	18.3	180.156
T2	Palmitic acid	Saturated fatty acid, common in beauty products.	57-10-3	6.2	256.4
	Pyrrole (1, 2, a) pyrazine 1, 4, dione, hexahydro 3- (2-methylpropyl)	Anticancer metabolite.	5654-86-4	17.3	210.273
T3	Silicic acid	Chemical compound with silicon, stimulates collagen production, increases hair strength and thickness, helps safely remove plaque with brushing, desiccant.	3555-45-1	2.4	96.11
	Pyrrole (1, 2, a) pyrazine 1, 4, dione, hexahydro 3- (2-methylpropyl)	Anticancer metabolite.	5654-86-4	17.3	210.273
T4	Quinoline	Organic compound whose derivatives have antiseptic, antibiotic, amebicide properties. Dyes in shades of blue, yellow, and red are extracted.	1613-34-9	4.0	129.16
	2-deoxy-D-galactose	Glucose analog, tumor growth inhibitor.	1949-89-9	15.5	164.0685
T5	DL arabinose	Monosaccharide used for the in vitro culture of various microorganisms.	20235-19-2	3.27	150.13
	Mannitol	Alcohol, sweetener, blood plasma substitute, diuretic.	69-65-8	5.23	182.172
	2-deoxy-D-galactose	Glucose analog, tumor growth inhibitor.	1949-89-9	18.3	164.0685
	Palmitic acid	Saturated fatty acid, common in beauty products.	57-10-3	20.893	256.4
	Oleic acid	Monounsaturated fatty acid, hypotensive.	112-80-1	22.931	282.47

## Data Availability

Not applicable.
